# Photoacid Generators for Biomedical Applications

**DOI:** 10.1002/advs.202302875

**Published:** 2023-12-01

**Authors:** Tianzhen Sun, Lin Kang, Hongyou Zhao, Yuxia Zhao, Ying Gu

**Affiliations:** ^1^ School of Medical Technology Beijing Institute of Technology No. 5 South Street, Zhongguancun Haidian District Beijing 100081 China; ^2^ Key Laboratory of Photochemical Conversion and Optoelectronic Materials Technical Institute of Physics and Chemistry Chinese Academy of Sciences No. 29 Zhongguancun East Road, Haidian District Beijing 100190 China; ^3^ University of Chinese Academy of Sciences No. 19A Yuquan Road Beijing 100049 China; ^4^ Department of Laser Medicine The First Medical Centre Chinese PLA General Hospital No. 28 Fuxing Road, Haidian District Beijing 100853 China

**Keywords:** antibacterial treatment, biomedical applications, photoacid generator, tumor treatment, water‐dependent reversible photoacid

## Abstract

Photoacid generators (PAGs) are compounds capable of producing hydrogen protons (H^+^) upon irradiation, including irreversible and reversible PAGs, which have been widely studied in photoinduced polymerization and degradation for a long time. In recent years, the applications of PAGs in the biomedical field have attracted more attention due to their promising clinical value. So, an increasing number of novel PAGs have been reported. In this review, the recent progresses of PAGs for biomedical applications is systematically summarized, including tumor treatment, antibacterial treatment, regulation of protein folding and unfolding, control of drug release and so on. Furthermore, a concept of water‐dependent reversible photoacid (W‐RPA) and its antitumor effect are highlighted. Eventually, the challenges of PAGs for clinical applications are discussed.

## Graphical abstract

With the growing demand for biomaterials, many PAGs have been designed and synthetized for biomedical applications. Here, the recent progresses of PAGs for tumor treatment is summarized, antibacterial treatment, regulation of protein folding and unfolding, control of drug release and so on. Furthermore, W‐RPA and its applications are highlighted. Finally, the challenges of PAGs for clinical applications are discussed.

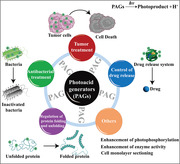

## 1 Introduction

Light has been used to treat diseases, such as psoriasis, rickets, vitiligo, smallpox and cutaneous tuberculosis, for more than three thousand years.^[^ ^1^ ^]^ Since Oscar Raab found that light can kill *Paramecium* in the presence of acridine in 1900, an increasing number of combinations of light and certain chemicals started to be developed and applied in the treatment of various diseases.^[^ ^2^ ^]^ At present, three types of photo‐triggered materials have been developed for biomedical applications. 1) Photosensitizers (PSs), which can be activated by light to generate reactive oxygen species (ROS).^[^ ^3^ ^]^ 2) Photothermal materials, which can convert the absorbed light energy into heat.^[^ ^4^ ^]^ 3) Photoacid generators (PAGs), which are compounds capable of producing hydrogen protons (H^+^) upon irradiation.^[^ ^5^ ^]^ Among of them, PSs mediated photodynamic therapy (PDT) has been used in clinic and achieved good therapeutic effects in the treatment of various diseases, especially in cancer treatment, such as skin cancer,^[^ ^2e^ ^]^ nasopharyngeal cancer,^[^ ^2f^ ^]^ breast cancer,^[^ ^6^ ^]^ and so on. However, the hypoxic environment of tumor seriously limits the efficacy of PDT.^[^ ^7^ ^]^ Photothermal materials mediated photothermal therapy (PTT) has also been widely studied in the biomedical field. However, the high temperature induced by PTT not only burns the lesion tissue but also damages nearby normal tissues accidentally. So far, none photothermal material has been approved to applied in tumor treatment.^[^ ^2d^ ^]^ Compared with PDT, PAGs mediated photoacid therapy (PAT) does not have the limitation of oxygen. And the acid production process of PAT is more controllable than the heat production process of PTT. Recently, with the growing demand for biomaterials in biomedical field, increasing number of PAGs with the specific properties have been designed and synthetized.

According to the mechanism of H^+^ production, PAGs are divided into irreversible and reversible PAGs.^[^ ^8^ ^]^ Irreversible PAGs are compounds that undergo irreversible photocleavage to produce H^+^ and by‐products,^[^ ^9^ ^]^ such as triarylsulfonium salts^[^ ^10^ ^]^ and trifluromethanesulfonic acids.^[^ ^11^ ^]^ By contrast, reversible PAGs are compounds that are capable of producing H^+^ under irradiation and taking the H^+^ back in dark, such as derivatives of naphthol^[^ ^8^ ^]^ and merocyanine.^[^ ^5d^ ^]^ In the past decades, PAGs have been widely used in polycondensations, cationic polymerization, positive photoresists,^[^ ^12^ ^]^ and some other H^+^ regulated events, such as controlling the intramolecular or intermolecular proton transfer,^[^ ^13^ ^]^ adjusting the volume of a hydrogels, and altering the conductivity of polymers upon irradiation.^[^ ^14^ ^]^

**Figure 1:**
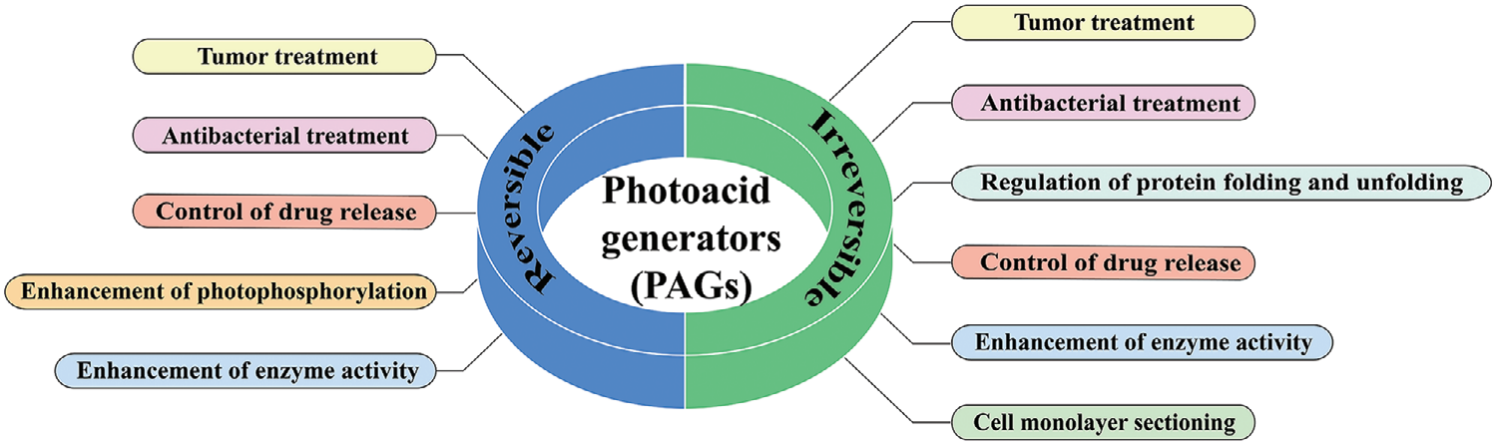
Schematic illustration of the applications of PAGs in biomedical field.

Herein, we systematically summarize the applications of irreversible and reversible PAGs in biomedicine, including tumor treatment, antibacterial treatment, regulation of protein folding and unfolding, control of drug release and so on (**Figure** **1**). Subsequently, a new concept of water‐dependent reversible photoacid (W‐RPA) and its applications in the tumor treatment are introduced. Eventually, the challenges and future perspectives of PAGs for biomedical applications are discussed.

## 2 Irreversible PAGs in Biomedical Applications

**Scheme 1:**
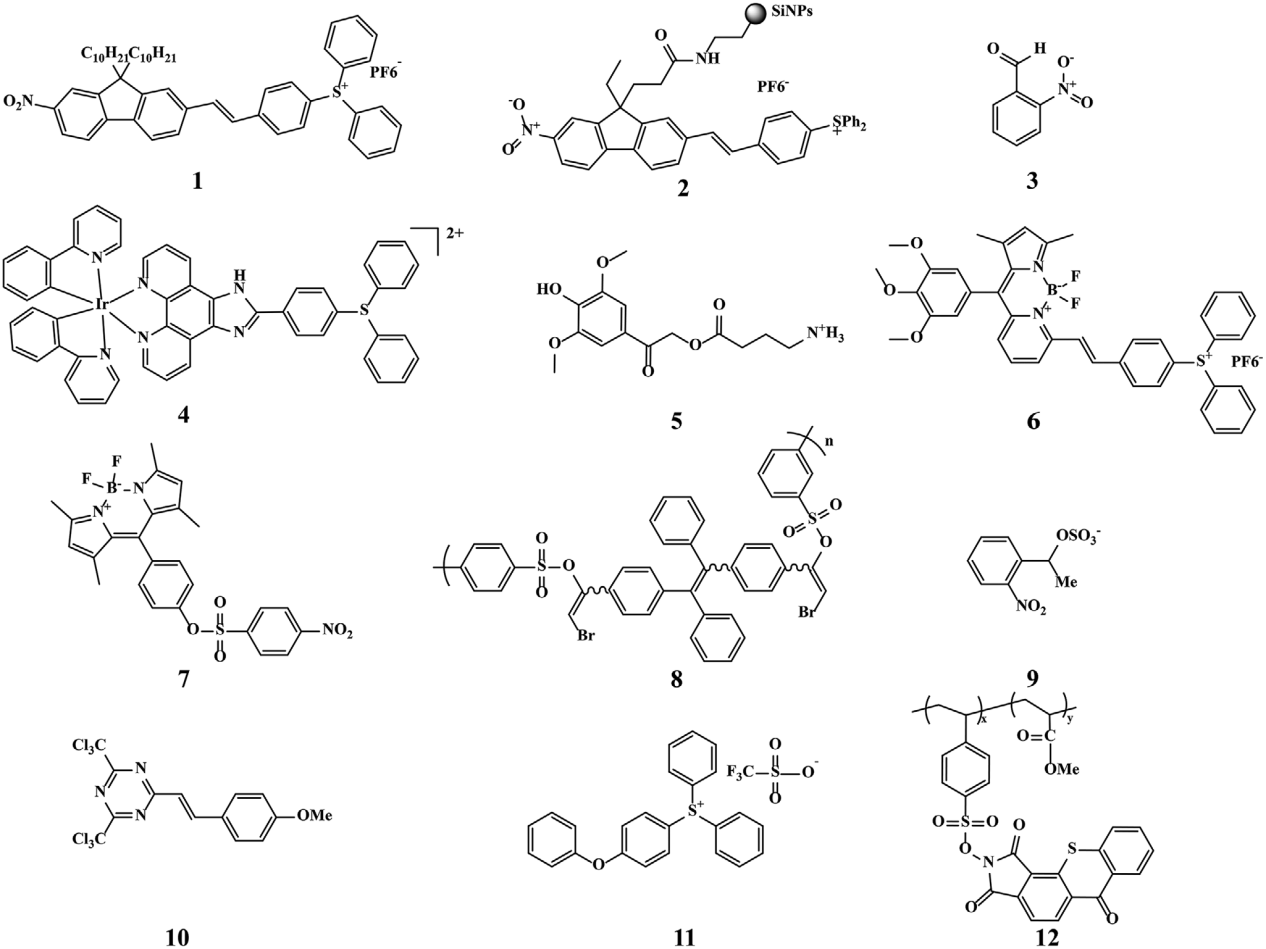
Chemical structures of irreversible PAGs for biomedical applications. 1, Triarylsulfonium salt derivative bonded with fluorene; 2, SiNP‐PAG9; 3, o‐NBA; 4, Triphenylsulfonium salt derivative bonded with Ir(III); 5, 4‐(2‐(4‐Hydroxy‐3,5‐dimethoxyphenyl)−2‐oxoethoxy)−4‐oxobutan‐1‐aminium; 6, BD‐PAG; 7, BDP‐S; 8, Polysulfonate; 9, 1‐(2‐nitrophenyl) ethyl sulfate (caged sulfate); 10, 2‐(4‐methoxystyrene)−4,6‐bis(trichloromethyl)−1,3,5‐triazine; 11, (4‐phenoxyphenyl) diphenylsulfonium triflate; 12, poly(methyl methacrylate).

The most well‐known irreversible PAGs used in the biomedical field are triarylsulfonium salt derivatives, such as compounds 1, 2, 4, 6, 11 in **Scheme** **1**. *o*‐Nitrobenzaldehyde (compound 3 in Scheme 1), 1‐(2‐nitrophenyl) ethyl sulfate (compound 9 in Scheme 1), sulfonic acid compounds (compounds 7, 8 in Scheme 1), n‐hydroxyphthalimide methacrylate (compound 12 in Scheme 1), carbon trichloride derivative (compound 10 in Scheme 1) are also classic irreversible PAGs. In recent years, these irreversible PAGs have been widely applied in tumor treatment, antibacterial treatment, regulation of protein folding and unfolding, control of drug release and so on.

### 2.1 Irreversible PAGs for Tumor Treatment

Tumor is the second largest cause of death in the world. ≈19.3 million new cancer cases and 10 million deaths from tumors were recorded globally in 2020.^[^ ^15^ ^]^ Therefore, novel tumor treatment modalities to complement existing therapies are urgently needed. Intracellular pH (pHi) plays an important role in the maintenance of cell physiological function.^[^ ^16^ ^]^ A slight variations of pHi will result in pathological changes in cells, such as the translocation of Bax from the cytosol to mitochondria^[^ ^17^ ^]^ and caspase activation.^[^ ^18^ ^]^ Subsequently, the cells will go to apoptosis. Tumor cells have been reported to have a weak acidic extracellular environment with a pH of 6.5–7.0 and a weak basic intracellular environment with a pH of 7.3–7.6.^[^ ^19^ ^]^ Several studies have reported that irreversible PAGs under light can kill tumor cells by lowering pHi and disrupting cellular functions, such as disrupting lysosomal function and inactivating key enzymes.^[^ ^20^ ^]^ At present, irreversible PAGs are applied in tumor treatment in two modalities: PAGs mediated PAT as a monotherapy kills tumor cells by producing H^+^ and lowering the pH of tumor cells^[^ ^20^, ^21^ ^]^ or PAGs mediated PAT combined with other antitumor strategies, such as PDT,^[^ ^21a^ ^]^ chemodynamic therapy (CDT),^[^ ^21b^ ^]^ and chemotherapy,^[^ ^21c,d^ ^]^ achieves synergistic antitumor effects.

#### 2.1.1 Irreversible PAGs Mediated PAT for Tumor Treatment

**Scheme 2:**
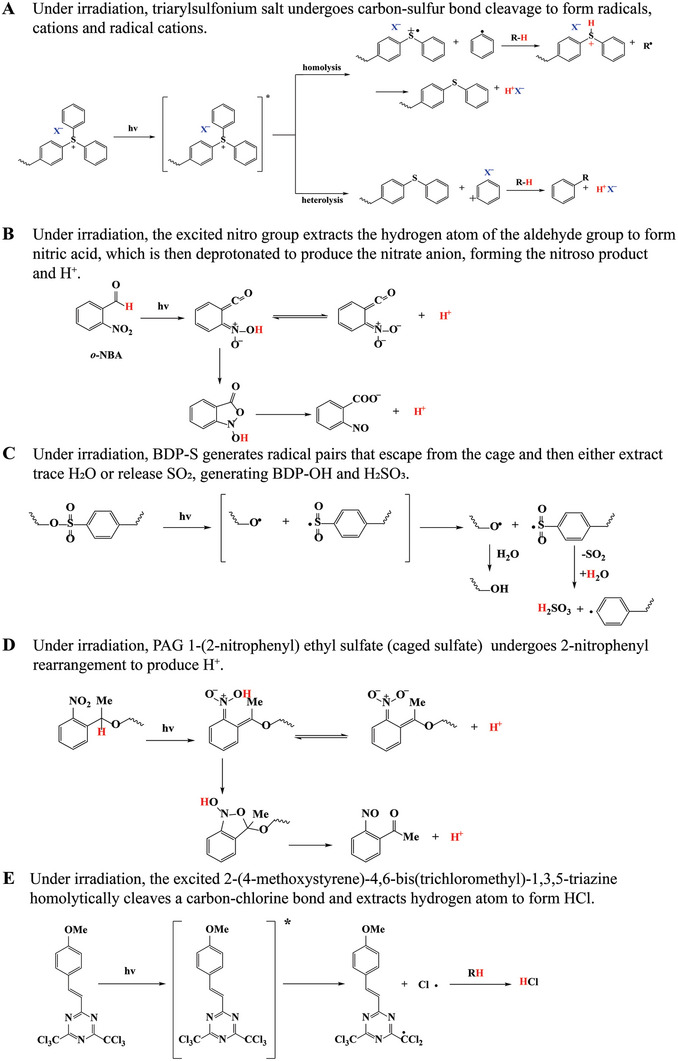
Photoreactions of irreversible PAGs for biomedical applications.

**Figure 2:**
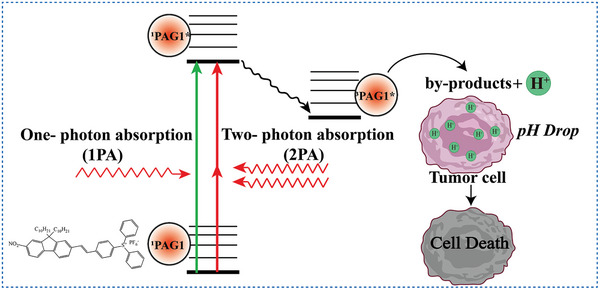
Schematic illustration of the PAG 1 inducing tumor cells death through 1PA or 2PA.

Triarylsulfonium salt derivatives are one of the most common irreversible PAGs. Under irradiation, triarylsulfonium salt undergoes carbon–sulfur bond cleavage simultaneously by two mechanisms (homolytic and heterolytic), resulting in the fragmentation of the cationic part of PAGs to form free radicals, cations, and radical cations. The subsequent reactions among these species, as well as between these species and solvent, produce a few final photolysis products (**Scheme** **2**A).^[^ ^9^ ^]^ In 2013, Belfield et al.^[^ ^20a^ ^]^ first proposed a novel strategy of PAT for tumor treatment. In this study, a triarylsulfonium salt derivative bonded with fluorene (compound 1, Scheme 1) as PAG (PAG1 in **Figure** **2**) was designed to be an efficient two‐photon absorbing molecule. Cell viability assays showed that PAG1 can significantly kill HCT 116 cells under 710 nm laser irradiation. Importantly, both one‐ and two‐photon absorption (1PA or 2PA) of the PAG can induce pHi imbalance and the death of tumor cells (Figure 2). In 2016, they further developed a polyethylene glycol (PEG)‐functionalized and hydrophilic silica nanoparticle (SiNP)‐enriched PAG (SiNP‐PAG9, compound 2 in Scheme 1). Similarly, 1PA or 2PA excitation of SiNP‐PAG9 can induce pHi drop and kill tumor cells.^[^ ^20b^ ^]^ These two studies not only demonstrated that PAT has a good antitumor effect in vitro, but also successfully applied 2PA technology in PAT.

**Figure 3:**
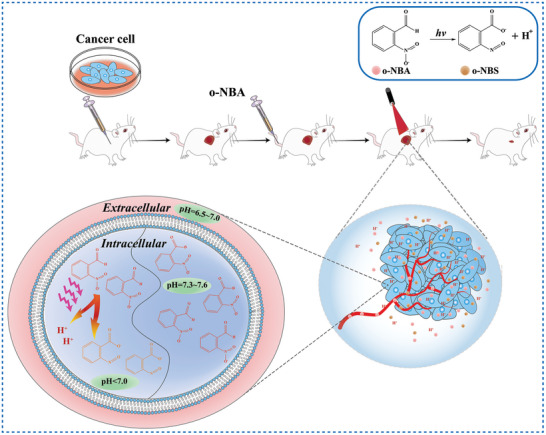
Schematic illustration of o‐NBA mediated PAT for tumor treatment under 405 nm laser irradiation.

Besides triarylsulfonium salt, *o*‐nitrobenzaldehyde (o‐NBA) is another widely used irreversible PAG. Under irradiation, the excited nitro group abstracts the hydrogen atom of the aldehyde group firstly to generate nitronic acid, which then deprotonates to yield nitronate anion, leading to the formation of nitroso product and H^+^ (Scheme 2B).^[^ ^22^ ^]^ In 2017, Gdovin et al.^[^ ^23^ ^]^ applied o‐NBA (compound 3, Scheme 1) in tumor treatment both in vitro and in vivo. The results showed that the photoactivation of o‐NBA with 405 nm laser significantly decreased the pHi of four tumor cell lines (MD‐MBA‐231, BxPC‐3, LNCaP, and 22Rv1) and exerted a significant killing effect on all these tumor cells in vitro. Additionally, o‐NBA mediated PAT caused significant reductions in tumor growth rate and enhanced the survival of mice bearing triple negative breast tumors. This study firstly demonstrates that PAT, as a monotherapy, has an antitumor effect both in vitro and in vivo. The mechanism of o‐NBA mediated PAT for tumor treatment is shown in **Figure** **3**.

#### 2.1.2 Irreversible PAGs Mediated PAT Combined with PDT for Tumor Treatment

**Figure 4:**
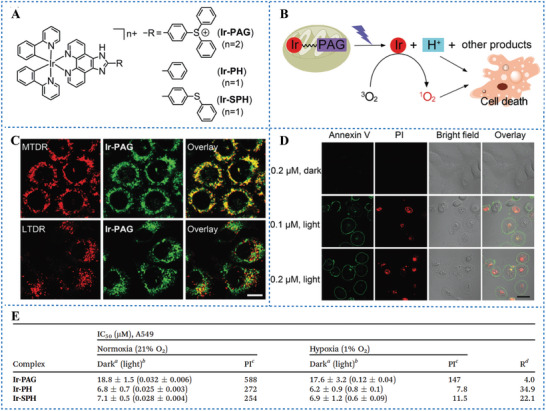
A) The chemical structures of Ir‐PAG, Ir‐PH and Ir‐SPH. B) Scheme of the phototherapeutic mechanism of Ir‐PAG. C) The co‐location images of Ir‐PAG with Mito Tracker Deep Red and Lyso Tracker Deep Red by confocal laser scanning microscopy (CLSM). Scale bar 20 µm. D) Detection of cell death in A549 cells co‐stained with annexin V‐FITC and PI by CLSM. Cells were incubated with different concentrations of Ir‐PAG with or without light. Scale bar 20 µm. E) Cytotoxicity of Ir‐PAG, Ir‐PH and Ir‐SPH on A549 cells under normoxic and hypoxic conditions. ^a^ Cells were treated with the tested complexes for 48 h. ^b^ Cells were treated with the tested complexes for 12 h before irradiation. ^c^ PI = IC_50_ (dark)/IC_50_ (light). ^d^ R = PI (normoxia)/PI (hypoxia). Reproduced with permission.^[^ ^21a^ ^]^ Copyright 2019, published by Royal Society of Chemistry.

PDT as a safe and effective treatment strategy has become a clinical choice for an increasing number of patients with tumor. Under irradiation, PSs transform neighboring molecular oxygen into highly cytotoxic ROS, eventually leading to tumor cell death.^[^ ^24^ ^]^ PS, O_2_, and light are the three essential elements of PDT. However, hypoxia occurs in most solid tumors due to the reduced O_2_ supply caused by disturbed microcirculation and deteriorated diffusion,^[^ ^25^ ^]^ which seriously limits the efficacy of PDT on solid tumors.^[^ ^26^ ^]^ By contrast, PAT is O_2_‐independent phototherapy. Therefore, the combination of PAT with PDT is a promising strategy for hypoxic tumor treatment. He et al.^[^ ^21a^ ^]^ designed and synthesized a mitochondria‐targeted dual‐mode phototherapeutic agent, Ir‐PAG (compound 4, Scheme 1), consisting of Ir(III) complexes combined with triphenylsulfonium salt (**Figure** **4**A). The lipophilic cationic Ir(III) complex has a high ^1^O_2_ quantum yield as an excellent PS and a high affinity to mitochondria (Figure 4C).^[^ ^27^ ^]^ Under 425 nm laser irradiation, Ir‐PAG also produces H^+^ and two major Ir (III)‐based photolysis products, Ir‐PH and Ir‐SPH. The results suggested that Ir‐PAG‐mediated PAT combined with PDT effectively damaged mitochondria and killed tumor cells through the production of H^+^ and ^1^O_2_ under normoxic or hypoxic condition (Figure 4B,D,E).

#### 2.1.3 Irreversible PAGs Mediated PAT Combined with CDT for Tumor Treatment

**Figure 5:**
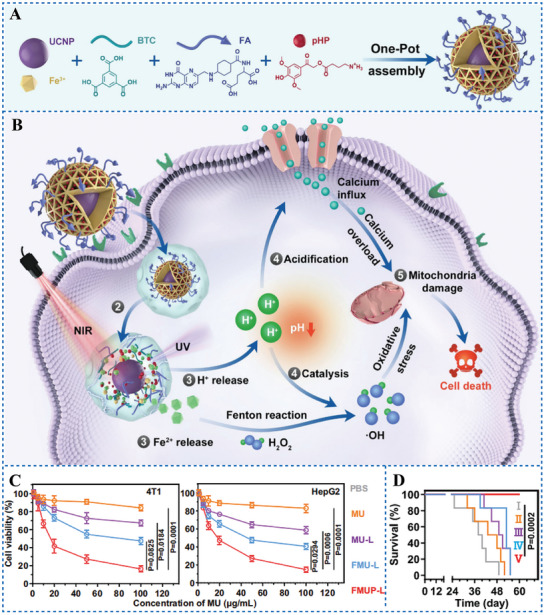
A) Schematic illustration of the construction of FMUP nanoagent. B) The underlying antitumor mechanism of FMUP nanoagent. C) CCK‐8 cytotoxicity analysis of 4T1 and HepG2 cells treated with PBS, MU (UCNP encapsulated in MOFs), MU‐L (MU with laser irradiation), FMU‐L, FMUP‐L. D) Survival curves of HeLa tumor‐bearing mice treated with different formulations. Reproduced under terms of the CC‐BY license.^[^ ^21b^ ^]^ Copyright 2021, published by Springer Nature.

CDT, a tumor treatment method based on the Fenton or Fenton‐like reactions, kills tumor cells by converting high concentrations of H_2_O_2_ into highly cytotoxic ·OH.^[^ ^28^ ^]^ Studies reported that ·OH can cause more severe oxidative stress and mitochondrial damage than ^1^O_2_.^[^ ^29^ ^]^ Given the advantages of low side effects, simple operation and endogenous activation, CDT has become an important research direction in tumor treatment.^[^ ^30^ ^]^ However, the Fenton reaction prefers an acidic environment with an optimal reaction pH range of 2.0–5.0,^[^ ^31^ ^]^ and the weak acidity of tumor cells is insufficient to induce an effective Fenton reaction for CDT.^[^ ^32^ ^]^ PAGs capable of reducing pH in tumor cells under irradiation may play a role in the tumor treatment of CDT. Following this hypothesis, Bao et al.^[^ ^21b^ ^]^ designed and synthesized a core‐shell type of nanoagent named FMUP, which had upconversion nanoparticles (UCNPs) in the core and coated with iron (III) carboxylate metal‐organic frameworks (MOFs). 4‐(2‐(4‐Hydroxy‐3,5‐dimethoxyphenyl)−2‐oxoethoxy)−4‐oxobutan‐1‐aminium (compound 5, Scheme 1) as PAG was encapsulated in the cavities of MOFs (**Figure** **5**A). UCNPs mediated the conversion of near‐infrared light (NIR) to ultraviolet (UV) excitation energy, and the generated UV subsequently mediated the reduction of Fe^3+^ to Fe^2+^.^[^ ^33^ ^]^ Simultaneously, PAG was activated and induced the acidification of intracellular microenvironment. The highly reactive Fe^2+^, acidic milieu, and H_2_O_2_ synergistically reinforced Fenton reactions to produce a large amount of ·OH (Figure 5B). The nanoagent significantly killed HeLa cells under irradiation in vitro. Moreover, it also effectively inhibited the tumor growth in HeLa tumor‐bearing mice and patient‐derived liver tumor xenograft models. Both in vitro and in vivo results suggested that the irreversible PAGs significantly improved the antitumor efficacy of CDT (Figure 5C,D). Therefore, the combination of irreversible PAGs mediated PAT and CDT is an effective antitumor strategy.

#### 2.1.4 Irreversible PAGs Mediated PAT Combined with Chemotherapy for Tumor Treatment

Chemotherapy has been the most widely used strategy to treat tumors in clinical settings.^[^ ^34^ ^]^ However, the resistance of tumor cells to chemotherapeutic drugs decreases the outcome of chemotherapy.^[^ ^35^ ^]^ Overcoming chemoresistance of tumor cells has become a challenge for tumor chemotherapy. Many studies have suggested that dysfunctional mitochondria play a critical role in chemoresistance.^[^ ^36^ ^]^ In normal cells, the mitochondrial membrane potential is −150 to −180 mV. In comparison, the mitochondrial membranes of tumor cells are hyperpolarized with a membrane potential as low as −220 mV, which promotes tumor cells migration and invasion.^[^ ^36^, ^37^ ^]^ In addition, membrane polarization directly affects mitochondrial outer membrane permeability and limits the release of cytochrome c (cyt c) and the activation of apoptotic pathways, subsequently causing the chemoresistance of tumor cells.^[^ ^37^, ^38^ ^]^ Belfield et al.^[^ ^21c^ ^]^ proposed a strategy to alleviate chemoresistance by modulating mitochondrial pH with PAGs. They used boron‐dipyrromethene (BODIPY) chromophore‐based triarylsulfonium PAG (BD‐PAG) to target mitochondria (compound 6, Scheme 1).^[^ ^39^ ^]^ Under 600 nm laser irradiation, the C─S bond of BD‐PAG breaks and generates H^+^.^[^ ^40^ ^]^ Acidified mitochondria can lead to mitochondrial membrane depolarization and activation of apoptotic pathways. To determine whether BD‐PAG can relieve chemotherapy resistance, BD‐PAG was used in combination with the antitumor drug chlorambucil (Cbl), an FDA‐approved drug for the treatment of chronic lymphocytic leukemia. The results showed that the viability of tumor cells was significantly reduced after the combined treatment of BD‐PAG and Cbl, compared with the treatment of Cbl only. It indicated that photoactivation of PAG to reduce pHi significantly improved the antitumor efficiency of Cbl. Thus, the combination of irreversible PAGs mediated PAT and chemotherapy can be of great help in overcoming chemoresistance of tumor cells.

**Figure 6:**
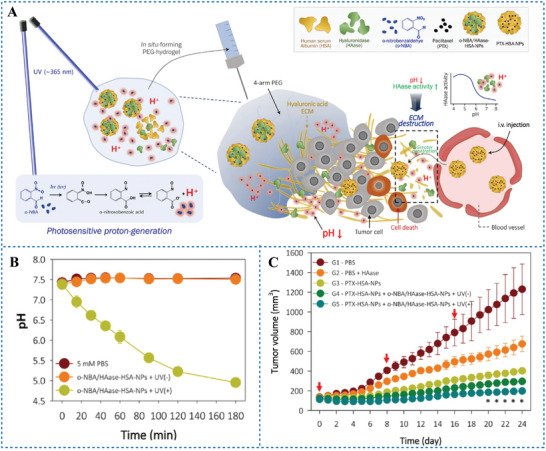
A) Schematic illustration of antitumor therapy with o‐NBA/HAase‐HSA‐NPs in conjunction with chemotherapeutic agents. B) Monitoring of the pH change induced by o‐NBA/HAase‐HSA‐NPs with or without light. C) The change of tumor volumes in five mice treatment groups over 24 days. Reproduced under terms of the CC‐BY license.^[^ ^21d^ ^]^ Copyright 2021, published by Elsevier.

Remodeling of the tumor extracellular matrix (ECM) has been reported to enhance the efficacy of chemotherapeutic agents.^[^ ^41^ ^]^ Hyaluronic acid (HA), one of the significant components of the ECM, overexpresses in most tumor cells and hinders the diffusion of chemotherapeutic agents.^[^ ^42^ ^]^ Hyaluronidase (HAase) can degrade HA and improve the diffusion of chemotherapeutic drugs and the efficacy of tumor treatment.^[^ ^42^, ^43^ ^]^ However, HAase is only active under acidic condition at around pH 4.5−5.5.^[^ ^44^ ^]^ In this situation, PAGs may enhance the activation of HAase by lowering the pHi. Lee et al.^[^ ^21d^ ^]^ prepared albumin NPs (o‐NBA/HAase‐HSA‐NPs) co‐loaded with o‐NBA and HAase (**Figure** **6**A). Under 365 nm laser irradiation, o‐NBA releases H^+^ and decreases the pH to 5.0 (Figure 6B). In this environment, HAase can be activated to enhance the degradation of HA and remodeling of ECM, ultimately improving the diffusion of chemotherapeutic agents and the efficacy of tumor treatment. The NPs combined with intravenous administration of the chemotherapeutic drug paclitaxel significantly enhance tumor suppression in AsPC‐1 tumor‐bearing mice (Figure 6C). These studies suggested that irreversible PAGs mediated PAT combined with chemotherapy are more effective in tumor treatment. Besides of the mitochondria and HAase, more potential targets of PAGs that can improve the antitumor efficacy of chemotherapy need to be explored.

### 2.2 Irreversible PAGs for Antibacterial Treatment

**Figure 7:**
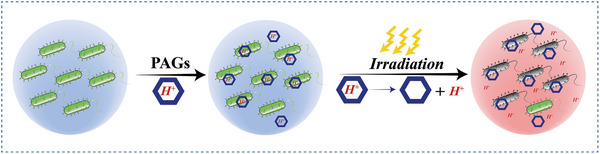
The procedure of PAGs produce H^+^ and kill bacteria.

In the past decades, antibiotic abuse has caused serious bacterial resistance, resulting in the emergence of drug‐resistant bacteria.^[^ ^45^ ^]^ The rapid increase of pathogenic microorganisms with antibiotic resistance severely threatens the lives of patients with infection. Therefore, developing a non‐pharmacological regimen for these patients is a top priority.^[^ ^46^ ^]^ Previous studies reported that lowering acidity can inhibit the synthesis of intracellular proteins and inactivate bacteria.^[^ ^47^ ^]^ Thus, PAT is a potential strategy to kill bacteria by lowering the pH of bacteria's living condition (**Figure** **7**).

#### 2.2.1 Irreversible PAGs Mediated PAT for Antibacterial Treatment

Sulfonate compounds are a class of irreversible PAGs, which undergo homolytic O─S bond cleavage to generate phenoxyl and sulfonyl radical pairs under irradiation. Ali et al.^[^ ^48^ ^]^ reported a fluorescence‐responsive PAG, boron dipyrromethene‐conjugated sulfonate (BDP‐S) (compound 7, Scheme 1). The generated radical pairs escape from the cage and abstract trace water or release SO_2_ to produce the BDP‐OH and H_2_SO_3_ (Scheme 2C). Therefore, BDP‐S was able to produce a large amount of H^+^ under white LED light irradiation, which dropped the pH from 7.4 to 4.6 and significantly reduced the number of survived *Escherichia coli (E. coli*). This result suggests that BDP‐S has the potential to be an excellent photoacid bactericide. Liu et al.^[^ ^49^ ^]^ developed photoresponsive polysulfonate (compound 8, Scheme 1) as a strong PAG to achieve UV‐triggered and irreversible broad‐spectrum sterilization. Gram‐positive *Staphylococcus aureus* (*S. aureus*), Gram‐negative *E. coli*, and *Pseudomonas aeruginosa* treated with PAGs mediated PAT were all killed, achieving 100% irreversible bacterial killing.

#### 2.2.2 Irreversible PAGs Mediated PAT Combined with PDT for Antibacterial Treatment

**Figure 8:**
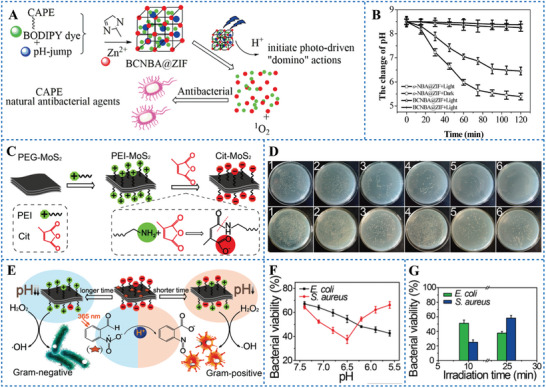
A) Schematic illustration of the synthesis and mechanism of BCNBA@ZIF nanoparticles. B) Monitoring of the pH change induced by o‐NBA and BCNBA@ZIF at different times under blue LED light or dark conditions. Reproduced under terms of the CC‐BY license.^[^ ^51^ ^]^ Copyright 2019, published by American Chemical Society. C) Schematic illustration of synthesis route of Cit‐MoS_2_(III). D) *E. coli* (up) and *S. aureus*(down) treated with 1, PBS; 2, 2‐NBA; 3, Cit‐MoS_2_(III); 4, H_2_O_2_; 5, o‐NBA + Cit‐MoS_2_(III); and 6, H_2_O_2_ + o‐NBA + Cit‐MoS_2_ (III), respectively. E) Scheme of the Gram‐selective antibacterial mechanism of Cit‐MoS_2_(III). F) Growth‐inhibition assay was conducted for *E. coli* and *S. aureus* in medium with different pH values. [Cit‐MoS_2_ (III)] = 90 µg mL^−1^. G) Growth‐inhibition assay was performed on Cit‐MoS_2_(III) (90 µg mL^−1^) and o‐NBA (600 µM) mixtures with different irradiation times. Reproduced under terms of the CC‐BY license.^[^ ^62^ ^]^ Copyright 2018, published by American Chemical Society.

PDT is not only an outstanding strategy for tumor treatment, but also widely used in antibacterial treatment.^[^ ^50^ ^]^ Lu et al.^[^ ^51^ ^]^ proposed a strategy of irreversible PAGs mediated PAT combined with PDT to improve antibacterial efficacy. They synthesized a photoactivated antibacterial platform, BCNBA@ZIF. It was obtained by coupling phenethyl caffeate (CAPE) with brominated BODIPY to synthesize BODIPY compound (BC) and then encapsulating BC and o‐NBA in the porous zeolitic imidazolate framework‐8 (ZIF‐8) which synthesized by 2‐methyl imidazole and Zn (II) ions (**Figure** **8**A). Under the irradiation of blue LED light, o‐NBA as a PAG produced H^+^, which dropped the pH from 8.5 to 5.5 (Figure 8B). The acidic environment allowed BC and Zn^2+^ to be released from ZIF8.^[^ ^52^ ^]^ BC as PS produces ^1^O_2_ under irradiation. The high concentration of Zn^2+^ also has specific toxicity.^[^ ^53^ ^]^ Finally, the combined therapy of PAT and PDT mediated by BCNBA@ZIF significantly inactivated *E. coli* through the production of H^+^, Zn^2+^, and ^1^O_2._ The antibacterial efficiency of BCNBA@ZIF under blue LED irradiation was almost equivalent to that of the clinical drug ampicillin.

#### 2.2.3 Irreversible PAGs Mediated PAT Combined with Chemotherapy for Antibacterial Treatment

To improve the antibacterial efficacy of chemotherapy, Song et al.^[^ ^54^ ^]^ synthesized a nanocomposite (RFP&o‐NBA@ ZIF‐8) by loading o‐NBA and the chemotherapy agent rifampicin (RFP) for antibacterial treatment into the mesopores of ZIF‐8. Under 365 nm laser irradiation, o‐NBA released H^+^, which induced the pH‐dependent degradation of ZIF‐8 to release Zn^2+^, followed by the release of RFP. Mice infected with methicillin‐resistant *S. aureus* (MRSA) or ampicillin‐resistant *E. coli* were treated with RFP&o‐NBA@ZIF‐8+light. The wounds of the mice were rapidly reduced by 80% with remarkable efficacy. This study demonstrated that irreversible PAGs mediated PAT combined with chemotherapy can effectively kill drug‐resistant bacteria in vivo.

#### 2.2.4 Irreversible PAGs Mediated PAT Combined with Peroxidase‐Like Metals Mediated Therapy for Antibacterial Treatment

Currently, nanozymes have been used in bio‐analysis,^[^ ^55^ ^]^ disease diagnosis,^[^ ^56^ ^]^ and treatment,^[^ ^57^ ^]^ and other fields.^[^ ^58^ ^]^ Peroxidase mimics, as one of the nanozymes, have been widely used to treat the wounds infected with bacteria because its capability of converting H_2_O_2_ to OH to kill bacteria.^[^ ^59^ ^]^ Metals such as Au, Ag, Pd and Pt have peroxidase‐like properties and have been used as peroxidase mimics in antibacterial treatment.^[^ ^60^ ^]^ However, the activity of peroxidase mimics is pH dependent, and most of them are inactive at near neutral pH.^[^ ^61^ ^]^ Therefore, PAGs have the potential to activate the peroxidase mimics due to their pHi‐reducing properties. Niu et al.^[^ ^62^ ^]^ synthesized PEI‐MoS_2_(II) through the electrostatic interactions between polyethylene glycol‐modified MoS_2_ and polyethyleneimine (PEI),^[^ ^63^ ^]^ and then obtained Cit‐MoS_2_(III) through forming an amide bond between citraconic anhydride (Cit) and ‐NH_2_ of PEI‐MoS_2_(II) (Figure 8C,D). Under 365 nm laser irradiation, the H^+^ produced by o‐NBA flexibly modulated the positivity and negativity of the surface charge of Cit‐MoS_2_(III), which selectively bound to Gram‐negative or Gram‐positive bacteria.^[^ ^64^ ^]^ Subsequently, the lower pH activated the peroxidase‐like activity of MoS_2_,^[^ ^65^ ^]^ which converted H_2_O_2_ to ·OH. The Gram‐positive *S. aureus* and Gram‐negative *E. coli* were effectively killed at 10 min of light exposure and 25 min of light exposure (Figure 8E–G). In vivo experiments showed that this strategy could effectively sterilize and promote wound healing of mice.

### 2.3 Irreversible PAGs for Regulation of Protein Folding and Unfolding

Proteins play an important role in all types of biological processes in all species of animals, plants, and microbes. The structure of proteins determines their function at the molecular level.^[^ ^66^ ^]^ The process by which proteins acquire their functional structure and conformation is known as protein folding. The mechanism of protein folding is a major unresolved biological problem in the central law of molecular biology.^[^ ^67^ ^]^ In their natural state, proteins remain folded due to the balance between their interactions with themselves and the environment. This balance can be disrupted by heat, cold, pressure, acids, bases, or organic denaturants.^[^ ^68^ ^]^ Acid‐induced unfolding and folding of myoglobin and its derivatives have been extensively studied.^[^ ^69^ ^]^ The rate of protein folding varies widely, from milliseconds to hours.^[^ ^70^ ^]^ The shortest observable time is ≈1 ms. Thus, changes in protein structure should be observed on nanosecond to microsecond time scales.^[^ ^71^ ^]^ Abbruzzetti et al.^[^ ^68f^ ^]^ first monitored the partial unfolding of apomyoglobin (ApoMb) triggered by a PAG‐induced pH jump via time‐resolved photoacoustic detection. ApoMb is formed by removing the heme moiety from myoglobin and has a secondary structure content and tertiary folding similar to that of myoglobin.^[^ ^72^ ^]^ Subsequently, the same team used o‐NBA to study the partial unfolding process of another protein, cyt c.^[^ ^73^ ^]^ Under acidic conditions, at least one of the ligands (His26 and His33) of GuHCl‐unfolded cyt c (cyt c Gu) was substituted by H_2_O.^[^ ^74^ ^]^ Importantly, they developed an instrumental setup that can monitor protein folding or unfolding processes by transient absorption. H^+^ produced by o‐NBA can exceed 100 µM in a few nanoseconds, which caused a rapid decrease in pH from neutral to about 4. For the first time, they achieved a jump of pH in nanoseconds, thereby providing a strategy for future studies on pH‐dependent protein folding and unfolding. Corrie et al.^[^ ^75^ ^]^ synthesized PAG 1‐(2‐nitrophenyl) ethyl sulfate (caged sulfate) (compound 9, Scheme 1), which underwent 2‐nitrophenyl rearrangement to produce H^+^ upon UV irradiation (Scheme 2D). The generated H^+^ triggered a decrease in pH from 4.6 to 4.2 and caused partial unfolding of a methemoglobin. Moreover, they proposed two requirements of PAGs applied to pH‐dependent protein unfolding driven by protonation of carboxylic acid side chains. First, the proton yield of PAG should be high enough to allow a pH below 4, and the reaction should be irreversible. Second, the proton release is rapid, and photolysis is efficient. In their subsequent studies, the complete unfolding of myoglobin under a single nanosecond laser pulse was induced by caged sulfate and observed for the first time.^[^ ^76^ ^]^ These studies confirmed that irreversible PAGs can be used to effectively regulate protein unfolding.

**Figure 9:**
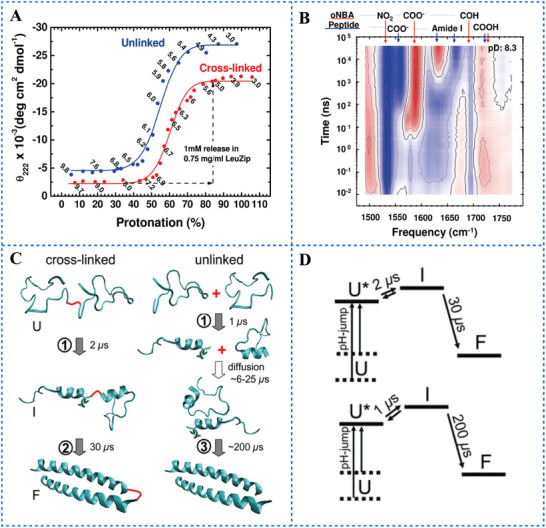
A) Plot of the folding of the leucine zipper versus the protonation of the cross‐linked (red) and unlinked (blue) peptide. B) Time resolved infrared difference spectrum showing early events associated with o‐NBA proton release, proton transfer, and the initiation of folding for cross‐linked peptide and starting pD 8.3 in the fully unfolded state. Three scans in different spectral windows were merged. The bands indicated by red arrows at the top of the spectrum are from o‐NBA and its photoproduct, and the bands indicated by blue arrows are from the leucine zipper. C) Sketch of leucine zipper folding for the cross‐linked (left) and unlinked peptides (right) from the unfolded (U) random coil crossing the partial α‐helical intermediate (I) to the folded state (F). D) A free energy model that can explain the observed kinetics. Reproduced under terms of the CC‐BY license.^[68g]^ Copyright 2018, published by American Chemical Society.

Besides of regulating protein unfolding, PAGs also play a role in the regulation of protein folding upon light irradiation. Causgrove et al.^[^ ^77^ ^]^ reported that the pH jump induced by o‐NBA was able to trigger the folding of poly‐L‐glutamic acid. Upon UV light irradiation, o‐NBA delivers H^+^ to the solution within 20 ns and causes a decrease of pH. The hydrogen proton neutralizes the carboxylate group of poly‐L‐glutamic acid, resulting in a reduction of Coulomb repulsion between the side chains of poly‐L‐glutamic acid and folding to form helix. The leucine zipper is a GCN4 mutant containing eight glutamate residues, which are pH sensitive.^[^ ^78^ ^]^ The pH conversion mechanism of the leucine zipper is essentially the same as that of poly‐L‐glutamate. Donten et al.^[68 g]^ investigated PAG‐induced folding of the leucine zipper. The protonation of covalently cross‐linked and unlinked peptides induced by o‐NBA was examined. The details of the leucine zipper changed from a completely unfolded state to a completely folded state were detected after 222 nm laser irradiation (**Figure** **9**A,B). Protein folding consists two steps: 1) 1–2 µs time scale for leading to partially folded α‐helix even in the case of monomers; 2) the time scales of 30 µs for the cross‐linked peptide and ≈200 µs for the unlinked peptide distinctively led to the final coiled‐coil structure (Figure 9C,D). These studies demonstrated that irreversible PAGs can regulate protein folding.

### 2.4 Irreversible PAGs for Control of Drug Release

**Figure 10:**
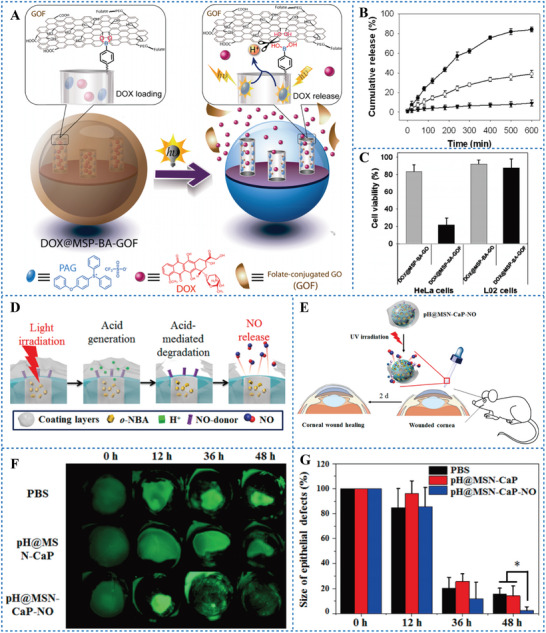
A) Schematic illustration of DOX@MSP‐BA‐GOF as a drug delivery system. B) DOX release profiles from DOX@ MSP‐BA‐GOF in aqueous solution after illumination for different times: 0 (black triangles), 5 (open circles), and 10 min (black circles). C) Cytotoxicity of DOX@MSP‐BA‐GOF and DOX@MSP‐BA‐GO incubated with HeLa cells and L02 cells for 24 h. Reproduced under terms of the CC‐BY license.^[^ ^88^ ^]^ Copyright 2014, published by American Chemical Society. D) Schematic illustration of the actuation principle of the o‐NBA for drug delivery system. E) Experimental protocol for corneal wound healing with pH@MSN‐CaP‐NO in vivo. F) Representative images of different groups of corneal wounds after staining with 0.5% fluorescein sodium solution. G) Quantification of wounded area after different groups of treatments. Reproduced under terms of the CC‐BY license.^[^ ^89^ ^]^ Copyright 2016, published by American Chemical Society.

The development of novel drug delivery systems (DDSs) is bringing a revolution to the pharmaceutical field.^[^ ^79^ ^]^ DDSs are macro/micro/nanocarriers that can carry drugs into the patient's body and significantly improve the accuracy of drug delivery.^[^ ^80^ ^]^ Drug release is the critical step of drug delivery, which determines the target site of drug. The controlled degradation of polymer particles triggered by external stimuli has received extensive attention in recent years.^[^ ^81^ ^]^ In particular, light stimulation offers the possibility for spatially and temporally controlled drug release because it is easily switched on/off in a confined area.^[^ ^82^ ^]^ For pH‐responsive micelles or nanoaggregates coloaded with drugs and PAGs can rapidly induce pH jumps and drug release. In this process, the PAGs like switches, which are flipped upon irradiation and the drug inside will be released. Techawanitchai et al.^[^ ^83^ ^]^ proposed a strategy to control pH‐responsive hydrogels for programmable drug release via o‐NBA. Under 365 nm laser irradiation, the o‐NBA‐loaded hydrogel releases a large amount of H^+^, which stimulates the massive release of drug in the hydrogels. The hydrogels provide a new research direction for designing predictive and programmable drug release devices. Park et al.^[^ ^84^ ^]^ synthesized polymeric microparticles consisting of a PAG named 2‐(4‐methoxystyrene)−4,6‐bis(trichloromethyl)−1,3,5‐triazine (compound 10, Scheme 1) and Ac‐Dex loaded with the antitumor drug irinotecan. Ac‐Dex is insoluble in water due to its high density of acetal groups. Conversely, acid‐hydrolyzed Ac‐Dex is soluble in water.^[^ ^85^ ^]^ Under 345 nm laser irradiation, the excitation of PAG led to the homolysis of one of the carbon‐chlorine bonds and the generation of HCl via hydrogen abstraction by the chlorine atom (Scheme 2E).^[^ ^86^ ^]^ The generated H^+^ allowed Ac‐Dex to dissolve in water and irinotecan to be released. Similarly, Wang et al.^[^ ^87^ ^]^ synthesized a PAG‐loaded micelle (P‐PD) by combining pH‐responsive polymeric micelle [poly (ethylene glycol)‐block‐poly(2‐(diisopropylamino) ethyl methacrylate), mPEG‐b‐PDPA] with PAG (compound 10, Scheme 1). Photochemical cleavage of the carbon‐chlorine bond in the PAG molecule produces a highly reactive chlorine radical. Under UV irradiation, it extracts a hydrogen atom from a solvent to produce HCl. Then the micelle‐loaded drug can be released in an acidic environment. P‐PD micelles co‐loaded with the antitumor drug doxorubicin (DOX) showed significant cell growth inhibition efficiency under irradiation. He et al.^[^ ^88^ ^]^ also designed a nanoplatform to control drug release by a PAG named (4‐phenoxyphenyl) diphenylsulfonium triflate (compound 11, Scheme 1). They immobilized PAG in mesoporous silica (MS) pores grafted with boric acid (BA) and then capped with folate‐conjugated graphene oxide (GO) to obtain NPs (MSP‐BA‐GOF). When DOX was loaded with MSP‐BA‐GOF, the released H^+^ from PAG under 254 nm laser irradiation broke the boroester bond to open the pore gate, allowing DOX to be released to induce tumor cell death (**Figure** **10**A,B). Their results showed that DOX@MSP‐BA‐GOF had a selective killing effect on tumor cells overexpressed folate receptors, while it had no significant effect on normal cells under irradiation (Figure 10C). Choi et al.^[^ ^89^ ^]^ designed a PAG‐responsive NO release system and applied it to treat corneal wounds of mice. o‐NBA and calcium phosphate (CaP) were loaded into mesoporous silica nanoparticles (MSN) to obtain pH@MSN‐CaP‐NO (Figure 10D). Under 365 nm laser irradiation, o‐NBA produced H^+^ and induced the degradation of CaP. The subsequent release of NO was used to heal the wound. After local administration of pH@MSN‐CaP‐NO in mice, a significant enhancement in wound healing and almost complete re‐epithelialization of corneal wounds were observed (Figure 10E–G). These studies suggested that PAGs provide a method of light controlled timing and location of drug release, which can significantly improve selectivity and reduce the side effects of drugs.

### 2.5 Irreversible PAGs in Other Biomedical Applications

In addition to the discussed aspects above, irreversible PAGs also have been applied in the enhancement of enzyme activity,^[^ ^90^ ^]^ and cell monolayer sectioning.^[^ ^91^ ^]^

The control of the initiation of biochemical events, especially enzyme activity, is of high interest for research in recent years.^[^ ^92^ ^]^ Light can mediate the activation of intrinsic photosensitive proteins, for example, green fluorescent proteins.^[^ ^93^ ^]^ Non‐photosensitive enzymes can be activated by light‐activated agents.^[^ ^94^ ^]^ Kohse et al.^[^ ^90^ ^]^ firstly used o‐NBA to activate non‐photosensitive acid phosphatase. Under irradiation at 388 nm, the released H^+^ from o‐NBA induced a pH jump of ≈3 units, allowing acid phosphatase to switch from inactive to active conditions. Therefore, the application of PAGs is an attractive approach to control enzyme activity. Sumaru et al.^[^ ^91^ ^]^ demonstrated that PAG (compound 12 in Scheme 1)‐induced pH imbalance can effectively kill adherent cells cultured on PAG‐loaded polymers under low‐intensity visible light irradiation. This method can cleave induced pluripotent stem cells (iPSC) and Madin–Darby canine kidney cell monolayers into cell clumps with the desired size.

## 3 Reversible PAGs in Biomedical Applications

**Scheme 3:**
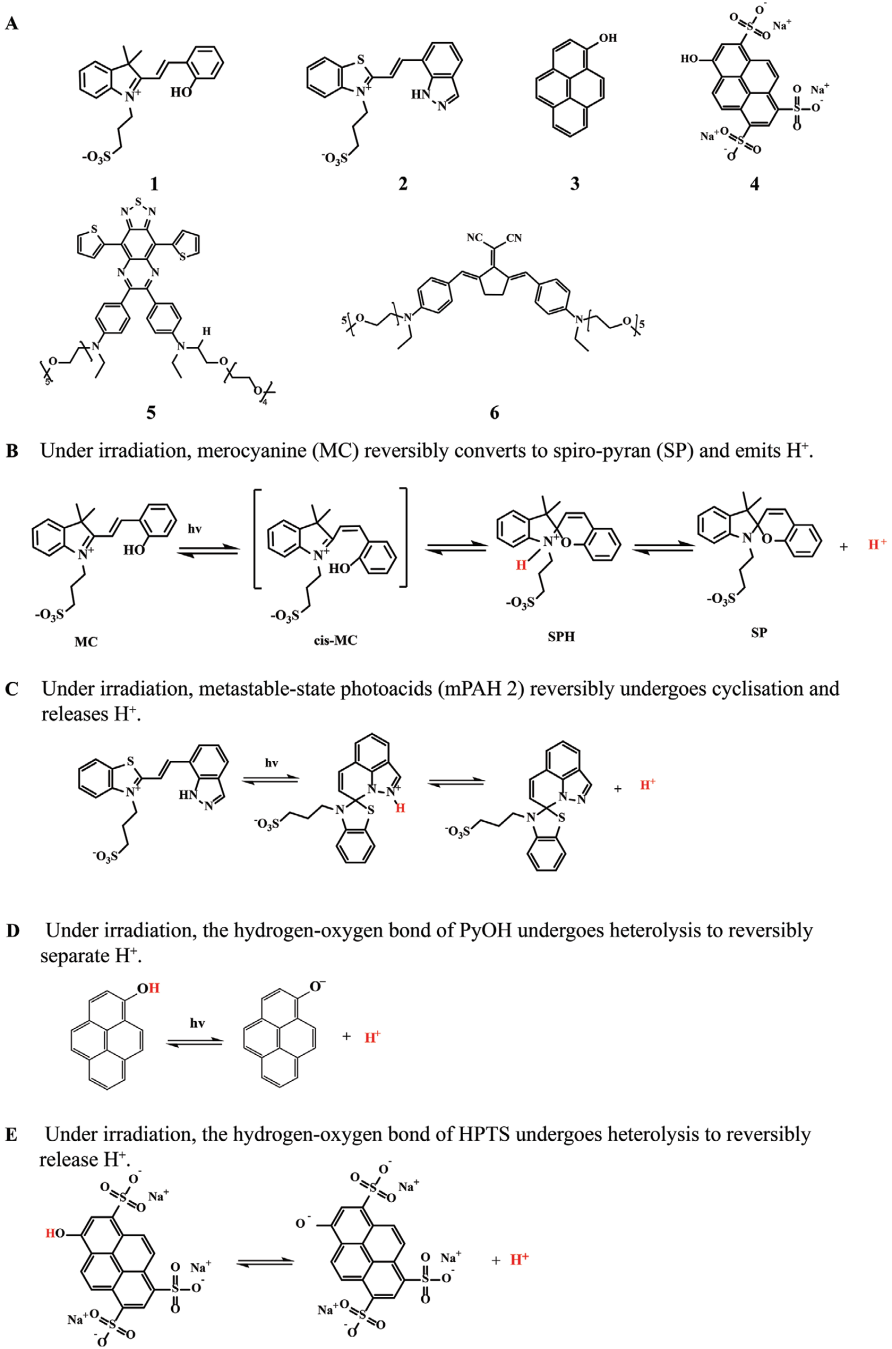
A) Structures of MC (compound 1), mPAH 2 (compound 2), PyOH (compound 3), HPTS (compound 4), TQs‐Th‐PEG5 (compound 5), BN‐PEG5 (compound 6). B) Proposed mechanism of the photoreaction of 1. C) Proposed mechanism of the photoreaction of 2. D) Proposed mechanism of the photoreaction of 3. E) Proposed mechanism of the photoreaction of 4.

The constant drop of pH induced by irreversible PAGs might lead to irreversible damages in cellular and life activities.^[^ ^95^ ^]^ Compared with irreversible photoacids, reversible photoacids neither produce by‐products nor intracellular H^+^ accumulation after light exposure, which may be safer for biomedical applications.^[^ ^8^, ^9^ ^]^ However, there are only a few reversible PAGs currently applied in biomedical field. Merocyanine (MC), compound 1 in **Scheme** **3**A, is the first reported and the most used irreversible PAG to date.^[^ ^13c^ ^]^ It was shown that MC can be reversibly converted to spiro‐pyran (SP) and emit light‐driven protons under irradiation (Scheme 3B).^[^ ^8^ ^]^ Based on the MC, compound 2 in Scheme 3A was synthesized, named as metastable‐state photoacids 2 (mPAH 2).^[^ ^96^ ^]^ The proposed mechanism of the photoreaction of mPAH 2 is shown in Scheme 3C. In addition, pyrenyl alcohol (compound 3 in Scheme 3) and its derivative (compound 4 in Scheme 3) are shown in Scheme 3.^[^ ^97^ ^]^ Recently, a concept of water‐dependent reversible photoacid (W‐RPA) was proposed. And two reversible PAGs (compound 5, compound 6 in Scheme 3) with W‐RPA property have been designed and synthesized.^[^ ^98^, ^99^ ^]^ These reversible PAGs have been applied in basic research for tumor treatment, antibacterial treatment, control of drug release and so on.

### 3.1 Reversible PAGs for Tumor Treatment

As classic reversible PAGs, MC and mPAH 2 have been applied in antitumor therapy in basic research.^[^ ^8^, ^96^ ^]^ However, both of them are mainly combined with other antitumor strategies, such as PDT and CDT.^[^ ^100^ ^]^

#### 3.1.1 Reversible PAGs Mediated PAT Combined with PDT for Tumor Treatment

Tumor cells are weakly basic inside, which is not favorable for acid‐responsive nanomaterials working inside tumor cells.^[^ ^101^ ^]^ Therefore, adjusting the intracellular microenvironment from weakly basic to weakly acidic is helpful to improve the efficiency of acid‐responsive nanomaterials in tumor treatment. Wang et al.^[^ ^100a^ ^]^ synthesized nanomaterials (UCNP@ZIF + TPP + PA) with the core of UCNPs covered by ZIF‐8, which were then co‐loaded with mPAH 2 and acid‐responsive porphyrin (TPP). Under 980 nm laser irradiation, the emitted light from UCNPs activated PAG to produce H^+^, which resulted in the decrease of the intracellular pH. Under acidic conditions, TPP was protonated (TPP‐H^+^), which reduced the aggregation of TPP and increased ^1^O_2_ production. Both in vitro and in vivo experiments demonstrated that PAG could significantly improve the efficacy of PDT.

#### 3.1.2 Reversible PAGs Mediated PAT Combined with CDT for Tumor Treatment

**Figure 11:**
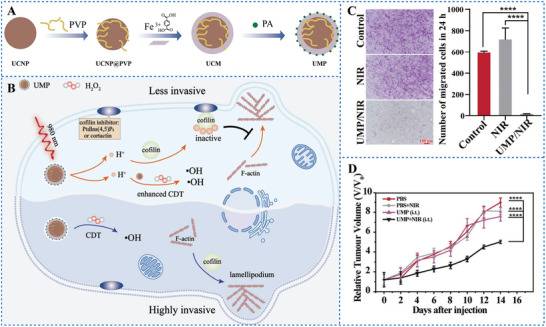
A) Schematic illustration of the synthesis of UMP nanoparticles. B) Scheme of UMP inhibition of tumor invasion and enhancement of CDT. C) Representative images of cell invasiveness in a Matrigel‐based transwell invasion assay. Scale bar 100 µm (left). D) Relative tumor volume changes during 14 days of treatment in U87MG tumor‐bearing BALB/c‐nu mice with different groups. Reproduced with permission.^[^ ^100b^ ^]^ Copyright 2021, published by German Chemical Society.

Tumor cells with infinite proliferation have the potential to invade adjacent tissues and vascular systems and eventually metastasize.^[^ ^102^ ^]^ More than 90% of cancer patients died from tumor metastasis. So anti‐metastasis is the point to prolong the patients’ life.^[^ ^103^ ^]^ However, the FDA‐approved antimetastatic drugs or methods are limited.^[^ ^104^ ^]^ Therefore, it is urgent to explore new antimetastatic therapies. Inhibition of the activity of cofilin, an actin‐binding protein, will lead to significant defects in cell invasion motility and directed protrusion formation, contributing to antimetastatic therapy.^[^ ^105^ ^]^ The low pH can induce cofilin protonation, and cause the inactivation of cofilin. Chen et al.^[^ ^100b^ ^]^ proposed an approach to enhance the antitumor efficacy of CDT by using PAG to inhibit cofilin activity. They designed a NIR‐controlled NP, named as UMP, with a core of UCNPs coated with MIL‐88B for internal loading of mPAH 2 (**Figure** **11**A). Under 980 nm laser irradiation, mPAH 2 raised the acidity of the environment, allowing iron, which acted as a catalytic active center in MIL‐88B, to initiate an enhanced Fenton reaction, culminating in enhanced CDT (Figure 11B). Thus, the combination of reversible PAG and CDT not only significantly improved the efficacy of killing tumor but also decreased the metastasis of tumor (Figure 11C,D).

### 3.2 Reversible PAGs for Antibacterial Treatment

**Figure 12:**
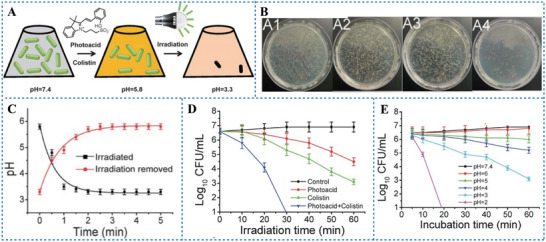
A) Schematic diagram of MC antibacterial. B) Cultured MDR *Pseudomonas aeruginosa* after treating with PBS (A1), colistin (A2), illuminated photoacid (A3), and colistin together with the illuminated photoacid (A4). C) Monitoring of the pH change induced by MC with or without light. D) The effect of irradiation time on the number of bacteria. E) The effect of different pH values on the number of bacteria. Reproduced with permission.^[^ ^95^ ^]^ Copyright 2013, published by Royal Society of Chemistry.

Colistin is an effective antibacterial drug, which kills bacteria by disrupting the bacterial outer membrane and causing small molecules excreted from the cytoplasm.^[^ ^106^ ^]^ However, due to its severe nephrotoxicity and neurotoxicity, colistin is the last choice for the treatment of multidrug resistance (MDR).^[^ ^107^ ^]^ To reduce the side effects caused by MDR, combined therapies are increasingly being considered. Luo et al.^[^ ^95^ ^]^ combined colistin with PAG to kill MDR *P. aeruginosa* (**Figure** **12**A). Under visible light irradiation at 470 nm, colistin combined MC killed 99% of MDR *P. aeruginosa* (Figure 12B–E). More importantly, MC reduced the minimum inhibitory concentration of colistin against MDR *P. aeruginosa* from 8 mg mL^−1^ to 0.25 mg mL^−1^.

For wound treatment, the pH range at the site of infection is 6.5–8.5; the higher pH, the more severe infection.^[^ ^108^ ^]^ Xi et al.^[^ ^109^ ^]^ proposed the strategy to activate peroxidase mimics by lowering the pH with MC. They synthesized copper sulfide NPs (CuS NPs). Under visible light irradiation, MC induced a decrease in pH at the wound site of mice, which activated the peroxidase‐like activity of CuS NPs and significantly accelerated the wound healing in mice. The wound closure rate of mice on the 11th day in the CuS/H_2_O_2_/MC/light treatment group was as high as 88.1%. Transcortical potential difference is an endogenous electric filed that has been shown to promote wound healing.^[^ ^110^ ^]^ Yin et al.^[^ ^111^ ^]^ reported a light‐powered dressing, which consisted of MC and electroactive polymer polyaniline (PANI). Under xenon lamp irradiation, the H^+^ produced by MC promoted the formation of the reduced state of PANI and the generation of photocurrents, which resulted in the formation of an electric field at the tissue interface. Photocurrent interacts with endogenous electric filed can promote skin growth. The acidic environment produced by MC significantly inhibited the growth of *E. coli* and *S. aureus*. Dorsal wounds in Male Sprague‐Dawley (SD) rats dressed with PANI/MC healed completely on day 14. Therefore, both in vitro and in vivo studies demonstrated that reversible PAG has a good prospect in the antibacterial field.

### 3.3 Reversible PAGs for Control of Drug Release

Like irreversible PAGs, reversible PAGs are also applied in control of drug release. Han et al.^[^ ^112^ ^]^ designed and fabricated MC‐assisted drug release NP based on mesoporous silica‐coated UCNPs. Under 980 nm laser irradiation, MC released H^+^, which promoted the cleavage of the acid‐labile cap and opened the pores. The antitumor drug DOX was released, resulting in the death of HeLa cells. This is the first study that applied reversible PAG to control drug release. Subsequently, Liu et al.^[^ ^113^ ^]^ developed a photoresponsive drug release system named UZPM, which consisted of UCNP@ZIF‐8, PAG, and melatonin (MT). MT can prevent the occurrence and progression of inflammation‐related depression. Under 980 nm laser irradiation, the blue light emitted by UCNP activated mPAH 2 to generate H^+^, triggering the destruction of ZIF‐8 to release MT. MT has certain immunomodulatory functions, but it is hard to pass through the blood–brain barrier.^[^ ^114^ ^]^ They addressed this issue by introducing UZPM into macrophages through functional liposomes fusion. Additionally, to precisely target central M1 type microglia, cytotoxic T‐lymphocyte‐associated protein‐4 was used as a chimeric antigen receptor (CAR) to modify the surface of macrophages. Finally, a cell membrane vaccine‐like system (CAR‐M‐UZPM) was then synthesized. Both in vitro and in vivo experiments showed that CAR‐M‐UZPM could effectively go through the blood–brain barrier and target M1‐type microglia, which could rapidly reduce the expression of pro‐inflammatory factors and prevent inflammation‐related depression.

### 3.4 Reversible PAGs in other Biomedical Applications

Reversible PAGs have also been used to enhance enzyme activity and photophosphorylation.^[^ ^97^, ^115^ ^]^ Moreno et al.^[^ ^116^ ^]^ prepared MC‐based enzyme nanoreactor to control enzymatic activity. Under Hg lamp irradiation, protons released by MC protonated nanoreactor’ polymersome membrane. This allowed the membrane to be permeable, so that the substrate can enter the nanoreactor and active the catalytic activity of the enzyme. Once irradiation stopped, protons were recovered and the membrane rapidly returned to an impermeable state, ultimately inhibiting the nanoreactor’ activity. Photophosphorylation is the process in which adenosine triphosphate (ATP) is synthesized in light by the cystoid membrane of plant chloroplasts or the chromophore of photosynthetic bacteria.^[^ ^118^ ^]^ The proton gradient is the driving force for ATP production by ATP synthase.^[^ ^119^ ^]^ To enhance the photophosphorylation process and provide more energy for photosynthetic life, artificial bionic systems constructed by PAGs have been proposed in recent years. Xu et al.^[^ ^117^ ^]^ reported a strategy to encapsulate MC into chloroplasts to form assembled natural‐artificial hybrids to enhance photophosphorylation. Compared with pure chloroplasts, the hybrids achieved ≈3.9 times greater ATP production and significantly improved photosynthetic efficiency. Xu et al.^[^ ^115^ ^]^ constructed an artificial multilayer protocell membrane with well‐organized structures to enhance photophosphorylation. The 1‐hydroxy pyrene (PyOH) molecule was selected as PAG (compound 3, Scheme 3). Under xenon lamp irradiation, the hydrogen‐oxygen bond of PyOH in the multilayer membrane underwent heterolysis to reversibly separate protons (Scheme 3D).^[^ ^97a^ ^]^ The proton gradient formed by the protons released from PyOH drives ATP synthase to produce ATP efficiently. Then their team proposed a strategy to synthesize artificially mimicked chloroplasts for ATP production.^[^ ^97b^ ^]^ 8‐Hydroxypyrene‐1,3,6‐trisulfonate trisodium salt (HPTS) was selected as PAG (compound 4, Scheme 3). Under irradiation, the hydrogen‐oxygen bond of HPTS undergoes heterolysis to reversibly separate H^+^ (Scheme 3E). Poly (allylamine hydrochloride) (PAH), HPTS, and recombinant ATP synthase liposomes were sequentially assembled by electrostatic interaction and formed a carrier for the proton gradient construct.^[^ ^120^ ^]^ H^+^ produced by HPTS under irradiation efficiently drives ATP synthase to produce ATP and greatly enhances the photophosphorylation reaction. Their studies are expected to be used to remotely control ATP consumption and enzyme catalysis.

### 3.5 Water‐Dependent Reversible Photoacid

**Figure 13:**
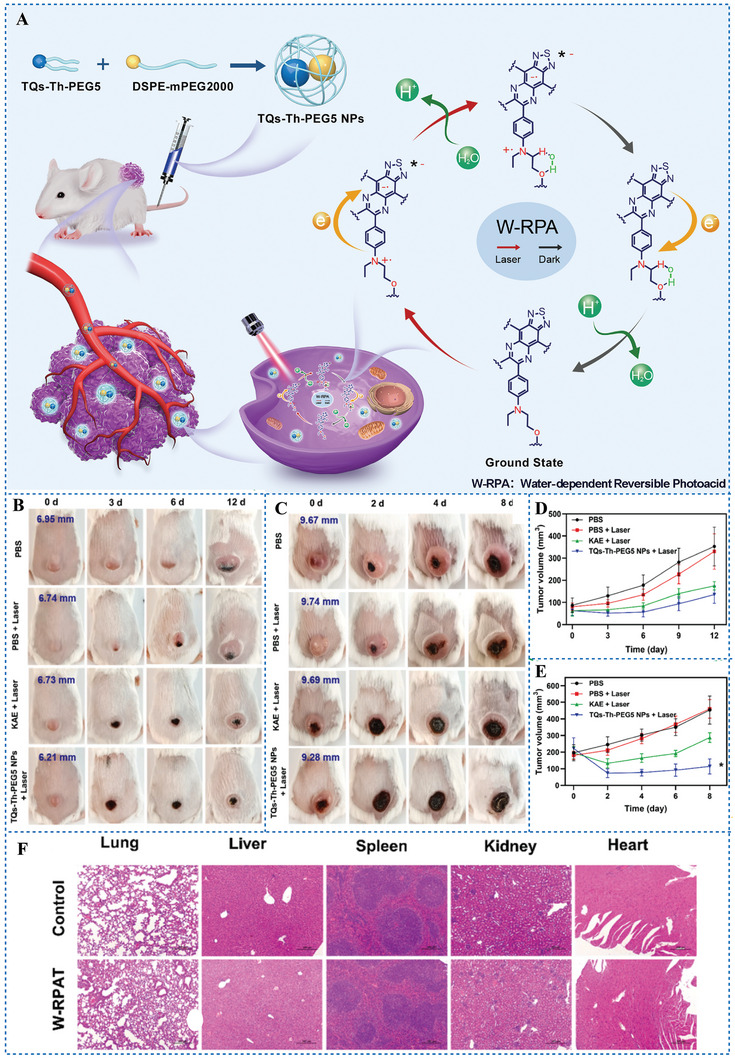
A) Schematic illustration of the mechanism of TQs‐Th‐PEG5 NPs mediated W‐RPAT for tumor treatment. B, C) Photographs of xenograft tumors in Balb/c mice treated by W‐PRAT with tumor diameter 6.0‐7.0 mm (B) and diameter 9.0‐10.0 mm (C), respectively. D, E) The change of tumor volumes after W‐RPAT corresponding to (B) and (C), respectively. F) H&E staining of major organs of tumor‐bearing Balb/c mice after one week of W‐RPAT. Reproduced with permission.^[^ ^98^ ^]^ Copyright 2022, published by Elsevier.

In 2022, Zhao et al.^[^ ^98^ ^]^ reported a novel mechanism of reversible photoacid named W‐RPA. Unlike previous PAGs, TQs‐Th‐PEG5, synthesized on the basis of thiadiazoloquinoxaline (TQ), cannot dissociate H^+^ by itself because it has no active hydrogen and no ability to form active hydrogen in its molecular structure. However, it is able to generate H^+^ reversibly under irradiation. The mechanism of W‐RPA is: under 660 nm laser irradiation, two hydrogen bonds are formed between the water and α‐H/γ‐O sites of TQs‐Th‐PEG5 forming a metastable six‐membered ring, then unlocked H atom in water dissociates in the form of H^+^. In the dark, the six‐membered ring dissociates and the produced H^+^ is reverted (**Figure** **13**A). Importantly, no oxygen is consumed in this process. TQs‐Th‐PEG5 NPs mediated W‐RPA therapy (W‐RPAT) exhibited significant antitumor efficacy both in vitro and in vivo (Figure 13B–E). The antitumor efficacy of W‐RPAT is better than that of KAE mediated PDT, especially in hypoxic tumors. Biosafety evaluation showed that TQs‐Th‐PEG5 NP mediated W‐RPAT has no adverse effects on living mice (Figure 13F). W‐RPA breaks through the limitation of traditional PAGs produce H^+^ must be excited by light with high energy.

**Figure 14:**
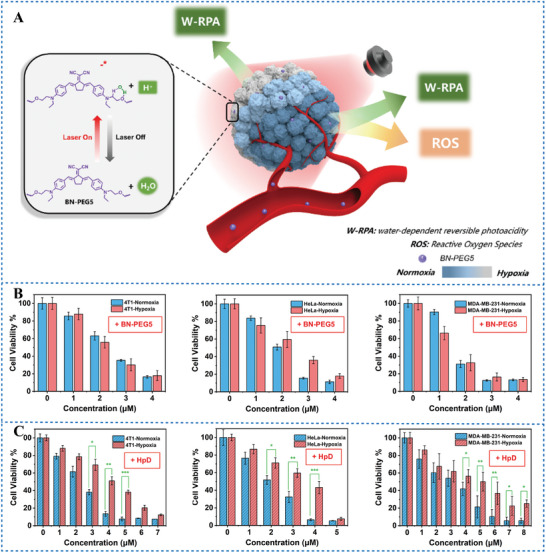
A) Schematic illustration of the mechanism of BN‐PEG5 with W‐RPA property for tumor treatment. B) Phototoxicity of BN‐PEG5 to different tumor cells under normoxia or hypoxia. C) Phototoxicity of HpD to different tumor cells under normoxia or hypoxia. Reproduced with permission.^[^ ^99^ ^]^ Copyright 2023, published by Elsevier.

Following the W‐RPA being proposed, Zhao et al.^[^ ^99^ ^]^ further developed a small molecule photosensitizer BN‐PEG5 based on cyclopentadienyl dinitrile. The mechanism of H^+^ production by BN‐PEG5 is the same as that of TQs‐Th‐PEG5. Under 635 nm laser irradiation, two hydrogen bonds are formed between the water and α‐H/γ‐O sites of BN‐PEG5 forming a metastable six‐membered ring, then unlocked H atom in water dissociates in the form of H^+^. Importantly, BN‐PEG5 has binary properties of W‐RPAT and PDT (**Figure** **14**A). Under a 635 nm laser irradiation, BN‐PEG5 can not only produce H^+^ reversibly but also produce ^1^O_2_. In vitro experiments have shown that BN‐PEG5 had efficient tumor treatment effects under either normoxic (≈20% O_2_) or extreme hypoxic (<5% O_2_) condition (Figure 14B). The therapeutic effect of BN‐PEG5 was not affected by O_2_ concentration, while the therapeutic effect of HpD under extreme hypoxic condition was significantly reduced (Figure 14C). Therefore, PAGs with binary properties of W‐RPAT and PDT can significantly improve the efficacy of tumour treatment under irradiation, especially for hypoxic tumours.

Classic reversible PAGs mediated PAT always combines with other strategies. In contrast, W‐RPAT has been applied as a monotherapy in tumor therapy and achieved remarkable antitumor efficacy. Although W‐RPAT is currently only used in tumor treatment, it has a great potential to be applied in other aspects of the biomedical field.

## 4 Conclusion and Perspective

PAGs with special light‐triggered properties have been increasingly applied in the biomedical field in recent years. In this review, we summarize the classification and biomedical applications of PAGs. Currently, PAGs are mainly applied in tumor treatment, antibacterial treatment, regulation of protein folding and unfolding, control of drug release and so on. The irreversible PAGs and reversible PAGs for biomedical applications are summarized in **Table** **1** and Table **2**.

**Table 1:** Irreversible PAGs for biomedical applications.

Applications		Selected PAGs	Compounds in Scheme 1	Effectiveness	References
Tumor treatment	Irreversible PAGs mediated PAT	sulfonium salt (PAG 1 and SiNP‐PAG9)	compound 1, compound 2	Induction of pHi imbalance and cell necrosis in HCT 116.	[20]
		o‐nitrobenzaldehyde (o‐NBA)	compound 3	Significant killing effects were observed against MD‐MBA‐231, BxPC‐3, LNCaP and 22Rv1. In vivo, it was able to improve the survival rate of tumor‐bearing mice.	[23]
	Combined with PDT	combination of Ir (III) complexes with triphenylsulfonium salts	compound 4	The combination of Ir‐PAG, Ir‐PH, and Ir‐SPH significantly killed A549 cells.	[21a]
	Combined with CDT	4‐(2‐(4‐hydroxy‐3, 5‐dimethoxyphenyl)−2‐oxoethoxy)−4‐oxobutan‐1‐aminium	compound 5	In vitro, significant killing of HeLa cells was achieved under irradiation. In vivo, significant efficacy was achieved in HeLa tumor‐carrying mice and patient‐derived liver tumor xenograft models.	[21b]
	Combined with chemotherapy	boron‐dipyrromethene (BODIPY) chromophore‐based triarylsulfonium	compound 6	Both HeLa and MCF‐7 cells was significantly killed after the combined treatment of BD‐PAG and Cbl.	[21c]
		o‐NBA	compound 3	Combined with the chemotherapeutic drug paclitaxel, it significantly inhibited the growth of tumors in AsPC‐1 tumor‐bearing mice.	[21d]
Antibacterial treatment		boron dipyrromethene conjugated sulfonate (BDP‐S)	compound 7	The pH decreased from 7.4 to 4.6, causing a significant decrease in *E. coli*.	[48]
		polysulfonates	compound 8	Achieved 100% irreversible bacterial killing of Gram‐positive *S. aureus*, Gram‐negative *E. coli* and *Pseudomonas aeruginosa*.	[49]
		o‐NBA	compound 3	The pH value decreased from 8.5 to 5.5, significantly inactivating *E. coli*.	[51]
		o‐NBA	compound 3	Wounds of mice infected with MRSA or ampicillin‐resistant *E. coli* were rapidly reduced by 80%.	[54]
		o‐NBA	compound 3	Better inhibits *E. coli* under weak acidic conditions and *S. aureus* under near neutral conditions. In vivo, it can effectively kill bacteria and promote wound healing in mice.	[62]
Regulation of protein folding and unfolding		o‐NBA	compound 3	Triggered the partial unfolding of ApoMb.	[68f]
		o‐NBA	compound 3	The dissociation kinetics of the non‐native axial heme ligand in GuHCl‐unfolded Fe (III) cyt c was investigated.	[73]
		1‐(2‐nitrophenyl) ethyl sulfate (caged sulfate)	compound 9	Methemoglobin was partial unfolded. Myoglobin was completely unfolding.	[75, 76]
		o‐NBA	compound 3	Triggering the folding of poly‐L‐glutamic acid.	[77]
		o‐NBA	compound 3	Leucine zipper from completely unfolded state to completely folded state.	[68 g]
Control of drug release		o‐NBA	compound 3	Enable predictable, timed and explosive drug release.	[83]
		2‐(4‐methoxystyryl)−4,6‐bis(trichloromethyl)−1,3,5‐triazine	compound 10	Capable of light‐controlled timed and local release of irinotecan, which significantly killed HT‐29 cells.	[84]
		2‐(4‐methoxystyryl)−4,6‐bis(trichloromethyl)−1,3,5‐triazine	compound 10	Demonstrated significant cell growth inhibition efficiency by co‐loading with the antitumor agent DOX.	[87]
		(4‐phenoxyphenyl) diphenylsulfonium triflate.	compound 11	Selective killing of tumor cells that overexpress the folate receptor.	[88]
		o‐NBA	compound 3	Significantly enhanced corneal wound healing and almost complete re‐epithelialization in mice.	[89]
Enhancement of enzyme activity		o‐NBA	compound 3	Acid phosphatase switched from the inactive to the active state.	[90]
Cell monolayer sectioning		poly (methyl methacrylate)	compound 12	Monolayers of human iPSC (201B7) and Madin‐Darby canine kidney (MDCK) cells were cut into cell clumps of desired size under irradiation.	[91]

**Table 2:** Reversible PAGs for biomedical applications.

Applications		Selected PAGs	Compounds in Scheme 3	Effectiveness	References
Tumor treatment	Combined with PDT	mPAH 2	compound 2	TPP was protonated with the help of PAG to produce ^1^O_2_ for enhanced photodynamic therapy in vivo and in vitro.	[100a]
	Combined with CDT	mPAH 2	compound 2	In vitro and in vivo, PAG promoted more ·OH production by tumor cells, achieving better anti‐tumor and anti‐metastatic therapy.	[100b]
Antibacterial treatment		MC	compound 1	Aided by PAG, a large amount of ·OH was produced to kill bacteria and significantly accelerated the rate of wound closure in mice in vivo.	[109]
		MC	compound 1	PANI/MC significantly inhibited the growth of *E. coli* and *S. aureus*.	[111]
Control of drug release		MC	compound 1	Release of the antitumor drug doxorubicin (DOX), resulted in significant HeLa cell death.	[112]
		mPAH 2	compound 2	It could cross the blood‐brain barrier and target M1 microglia to prevent the onset and progression of inflammation‐associated depression.	[113]
Enhancement of enzyme activity		MC	compound 1	Successful control of the initiation and shutdown of enzymatic reactions. It was confirmed by encapsulating glucose oxidase (GOx) and myoglobin (Myo) in the reactor for validation.	[116]
Enhancement of photophosphorylation		MC	compound 1	Approximately 3.9 times greater ATP production and significantly higher photosynthetic efficiency were observed.	[117]
		1‐hydroxy pyrene (PyOH)	compound 3	Drive the ATP synthase to produce ATP efficiently.	[115]
		8‐hydroxypyrene‐1,3,6‐trisulfonate trisodium salt (HPTS)	compound 4	Synthesized artificially mimicked chloroplasts achieved ATP production in vitro.	[97b]
Water‐dependent reversible photoacid		TQs‐Th‐PEG5	compound 5	Under irradiation, TQs‐Th‐PEG5 NPs exhibited significant antitumor efficacy in vitro and in vivo, especially in hypoxic tumors. The treatment effect is better than that of KAE mediated PDT.	[98]
		BN‐PEG5	compound 6	BN‐PEG5 had efficient tumor treatment effects under both normoxicand extreme hypoxic conditions in vitro.	[99]

The research on PAGs is currently making significant progress and breakthroughs in basic research. It is worth noting that different PAGs have their own advantages and disadvantages. Among of them, irreversible PAGs mostly have a higher rate of proton production, which may make them more effective than reversible PAGs to decrease the pH in some biomedical applications. However, irreversible PAGs usually produce a variety of uncertain by‐products through homolytic and heterolytic upon irradiation. These by‐products are not monolithic and these compositions are difficult to be identified in vivo.^[^ ^9^, ^121^ ^]^ The biosafety and biocompatibility of these complex by‐products have to be clearly studied before the clinical application. In contrast, reversible PAGs have no by‐products and no intracellular H^+^ accumulation after light exposure, which may be safer for biomedical applications. However, there are a fewer reversible PAGs available for biomedical applications.^[^ ^13c^ ^]^ In addition, both of irreversible PAGs and traditional reversible PAGs rely on high‐energy photon excitation. The recently proposed PAGs with W‐PRA properties break through the limitation of the conventional photoacid reaction relying on high‐energy photon excitation, but more PAGs with W‐RPA properties should be developed.

Herein, we list some unresolved issues and challenges of PAGs that need to be addressed before actual clinical application.

PAGs are mostly excited by UV or blue‐violet light with high energy (λ≤500 nm). However, UV light is harmful to human tissues. The penetration depth of light in this band is lower than 3 mm, which is only applied to treat superficial lesions. Upconverting NPs or two‐photon technology can improve the depth of PAT to a certain extent. Nevertheless, the efficiency of these two methods is low. Therefore, PAGs that can be effectively activated by near infrared light should be developed.

Although PAGs mediated PAT has been applied in biomedical field, the reaction mechanisms of PAT in different biomedical applications should be fully investigated, especially in tumor treatment. Furthermore, the differences of the reaction mechanisms between irreversible and reversible PAGs mediated PAT applied in tumor treatment also need further exploration.

Unlike PSs used by PDT in the clinic, which are mostly isolated from human blood (porphyrin) or plants (porphine), PAGs are derived from chemical synthesis, such as triarylsulfonium salt derivatives and merocyanine. The biocompatibility of most reported PAGs lacks systematic and comprehensive investigations. Thus, evaluating the long‐term biocompatibility of existing or newly synthesized PAGs can accelerate the clinical applications of PAGs.

For PAGs mediated PAT, the spatiotemporal distribution of H^+^ in the treatment area should be explored. The risk of H^+^ entering the vasculature and altering blood pH during PAT should be evaluated and clarified. For irreversible PAGs, whether the multiple photolytic products produced by PAGs during photochemical reactions are safe should be further studied.

With continuous efforts, exploration, and collaboration of researchers from different fields, including chemistry, materials, biology, pharmacology, and medicine, we believe more PAGs with good properties will emerge and PAT will benefit patients in the near future.

## Conflict of Interest

The authors declare no conflict of interest.
